# Treatment of Irregularity

**Published:** 1865

**Authors:** 


### TREATMENT OF IRREGULARITY.

Perhaps all who have ever attempted to correct irregularities of the teeth are familiar with the fact, that sometimes it is very difficult to retain a changed tooth in position till it becomes firm in its new place. Recently Dr. Blount, of Springfield, Ohio, described to us a method he has employed to accomplish this object. It consists in simply drilling a small hole in the palatine surface of the tooth to be retained, and a similar hole in an adjoining tooth, if it be firm, and if not, the nearest one that is firm; and into these holes put a little staple, and pack gold foil in the holes round it, to hold it firmly in place; this is worn till the tooth becomes firmly set in position. After this, when the staple is removed, the holes should be plugged. Of course, this should only be resorted to when other means will not readily accomplish the object. A good judgment will direct in all such matters.

